# Non-medical exemptions and state-level variation in kindergarten MMR vaccination coverage, United States, 2016–2024

**DOI:** 10.3389/fpubh.2026.1795927

**Published:** 2026-04-30

**Authors:** Diego R. Hijano

**Affiliations:** 1 Department of Infectious Diseases, St. Jude Children’s Research Hospital, Memphis, TN, United States; 2 Department of Pediatrics, University of Tennessee Health Science Center, Memphis, TN, United States

**Keywords:** measles-mumps-rubella (MMR), non-medical exemptions, pediatric population, public health policy, school-entry requirements, vaccination coverage, vaccination mandates

## Abstract

**Background:**

High kindergarten vaccination coverage is essential to sustain herd immunity against measles and other vaccine-preventable diseases. In recent years, rising state-level non-medical exemption rates and pandemic-related disruptions have threatened this protection in the United States.

**Methods:**

We analyzed 8 years of Centers for Disease Control and Prevention (CDC) kindergarten vaccination surveillance data (2016–2017 through 2023–2024) to assess national and state-level trends in vaccination coverage and exemption rates. Descriptive analyses and scatterplots were used to examine patterns across years. A cross-sectional state-level linear regression model using 2024–2025 data (*N* = 39 jurisdictions) was used to quantify the association between exemption prevalence and MMR vaccination coverage.

**Results:**

National MMR coverage declined from 96.0% in the 2018–2019 school year to 92.7% in 2023–2024, while exemption rates increased from 2.5% to 3.3%. Updated 2024–2025 national estimates indicate that coverage remained near 92.5% and exemption rates increased further to 3.6%. In a cross-sectional analysis of 2024–2025 data, each 1% increase in exemption prevalence was associated with an approximately 1.1 percentage-point decrease in MMR coverage (*p* < 0.001), with exemption prevalence accounting for approximately two-thirds of observed variation in coverage. States permitting broad non-medical exemptions, including Idaho and Alaska, consistently reported coverage below the 95% herd immunity threshold, whereas states with more restrictive exemption policies maintained near-universal coverage.

**Conclusion:**

Non-medical exemptions are strongly associated with state-level differences in kindergarten vaccination coverage in the United States. These findings suggest that exemption policies may play an important role in shaping population-level protection, although causal relationships cannot be established from this analysis.

## Highlights

- Non-medical exemptions are strongly associated with lower kindergarten vaccination coverage in the United States.
- Differences in exemption prevalence align with variation in vaccination coverage across states, highlighting the importance of policy environments in shaping population-level protection.

## Introduction

Vaccines remain among the most effective public health interventions ever developed. In the United States, the Vaccines for Children (VFC) program, established in 1994 to provide free vaccines to eligible children, has delivered over 71 billion doses and prevented more than 500 million illnesses, 32 million hospitalizations, and more than 1.1 million deaths (1). Economic evaluations have consistently demonstrated that the U.S. childhood immunization program yields substantial net economic benefits. Zhou et al. conducted a comprehensive economic evaluation of routine childhood immunization for the 1994–2013 birth cohorts and estimated that the program required approximately $107 billion in vaccination costs but generated $402 billion in direct medical savings and $1.5 trillion in societal benefits (2). More recent analyses have confirmed and expanded these findings, including updated CDC estimates for the 1994–2023 birth cohorts reporting approximately $540 billion in direct medical savings and $2.7 trillion in societal benefits, with additional cohort-specific analyses demonstrating similarly substantial economic value (3, 4). Together, these analyses demonstrate that the U.S. childhood immunization program remains both a highly effective public health intervention and one of the most economically valuable investments in preventive healthcare. By maintaining high vaccination coverage, these programs have nearly eliminated many childhood diseases and protected communities through herd immunity (5).

In recent years, however, this protection has weakened. Global vaccination coverage has stalled, and in the United States, multiple factors, including pandemic disruptions, declining public confidence, and increasing non-medical exemptions, have eroded herd immunity (6, 7). All states require vaccines for school entry, but 45 states permit non-medical exemptions for religious or philosophical reasons (8). The structure and accessibility of these exemptions vary substantially across states and are defined by state statutes and administrative policies. In some jurisdictions, parents seeking an exemption must complete formal documentation, obtain notarized signatures, submit annual renewal forms, or participate in educational counseling with a healthcare provider regarding the risks of vaccine-preventable diseases. Other states require only a signed parental statement or allow exemptions to be filed through simplified administrative processes with minimal verification. Several states—including California, Maine, New York, Connecticut, and West Virginia—have eliminated most or all non-medical exemptions following major outbreaks, while others continue to permit both religious and philosophical exemptions with relatively limited procedural barriers. These differences in legal structure and administrative requirements influence how easily exemptions can be obtained and contribute directly to interstate variation in vaccination coverage. As a result, school-entry immunization policies create a heterogeneous policy environment in which children’s vaccination status is shaped not only by parental preference but also by the regulatory framework governing exemptions in each state.

The connection between exemptions and outbreaks has been repeatedly documented. California’s elimination of non-medical exemptions following the 2015 Disneyland measles outbreak led to a sustained rise in vaccination coverage (9, 10). Conversely, states with broad non-medical exemption policies, such as Idaho, Alaska, and Texas, report persistently lower measles–mumps–rubella (MMR) vaccination coverage, often below 90% (7). In 2025, the United States experienced its largest measles resurgence in over 30 years, with more than 2,200 confirmed cases across 45 jurisdictions and multiple deaths (11). Over 90% of those affected were unvaccinated, illustrating the tangible risk of policy permissiveness. This resurgence has continued into 2026, with more than 1,200 confirmed measles cases reported by early March across more than 30 jurisdictions, the majority associated with ongoing outbreaks and occurring predominantly among unvaccinated individuals (11).

While vaccine hesitancy and misinformation remain important barriers, state-level exemption laws are modifiable and enforceable levers for public health protection. Small changes in exemption rates can have outsized epidemiologic effects: because measles requires vaccination coverage above 95% to prevent transmission, even a 5% exemption rate can undermine community immunity (12). Despite this, few studies have integrated multi-year state-level data to evaluate how exemption patterns quantitatively relate to vaccination coverage in the post-pandemic era.

This study uses 8 years of Centers for Disease Control and Prevention kindergarten surveillance data (2016–2017 through 2023–2024) to describe trends in vaccination coverage and exemptions. We also use cross-sectional 2024–2025 state-level data to quantify the association between exemption prevalence and MMR vaccination coverage and to assess how state policy environments relate to population-level protection (7). By presenting these results through visual analytics, we highlight how differences in exemption policy environments may contribute to variation in community-level protection.

## Methods

### Data source

We analyzed publicly available kindergarten vaccination surveillance data from the Centers for Disease Control and Prevention (CDC) for the 2016–2017 through 2023–2024 school years (7). These data are collected annually through the CDC School Vaccination Assessment Program (SchoolVaxView) using reports submitted by state and selected local health departments. The dataset includes state-level counts of kindergarten enrollment, vaccination coverage for required antigens, and reported medical and non-medical exemptions.

For the 2024–2025 school year, cross-sectional state-level data were available for 39 jurisdictions after excluding provisional and missing values and were used for the regression analysis. Weighted national estimates were used to provide updated descriptive context but were not included in the regression analysis.

Details of the regression specification are described in the Analysis section, with full diagnostics and outputs provided in the Supplementary materials.

This study used publicly available, de-identified surveillance data and did not require institutional review board approval.

### Key variables

Vaccination coverage was defined as the percentage of kindergarten children who were up to date for each required antigen, including measles–mumps–rubella (MMR), diphtheria–tetanus–acellular pertussis (DTaP), polio, hepatitis B, and varicella. Exemptions were defined as the percentage of kindergarteners with at least one documented vaccine exemption and were categorized as medical or non-medical, including religious and philosophical exemptions, as reported by states. Analyses were conducted at the state level by school year.

### Analysis

A cross-sectional linear regression model was used to quantify the association between exemption prevalence and MMR vaccination coverage using 2024–2025 state-level data. The analytic sample included 39 jurisdictions. The analytic unit was the jurisdiction.

The regression employed a simple ordinary least squares (OLS) specification with no additional covariates. Because the analysis was cross-sectional, year fixed effects were not applicable. Each jurisdiction contributed a single observation; therefore, clustering adjustments were not performed. No weighting was applied, as the analysis focused on the observed relationship across jurisdictions rather than generating nationally representative estimates.

Descriptive analyses and visualizations using multi-year data (2016–2017 through 2023–2024), including pooled state-year scatterplots, were conducted separately to examine longitudinal trends.

## Results

### National vaccination coverage has declined modestly but persistently

**Figure 1:**
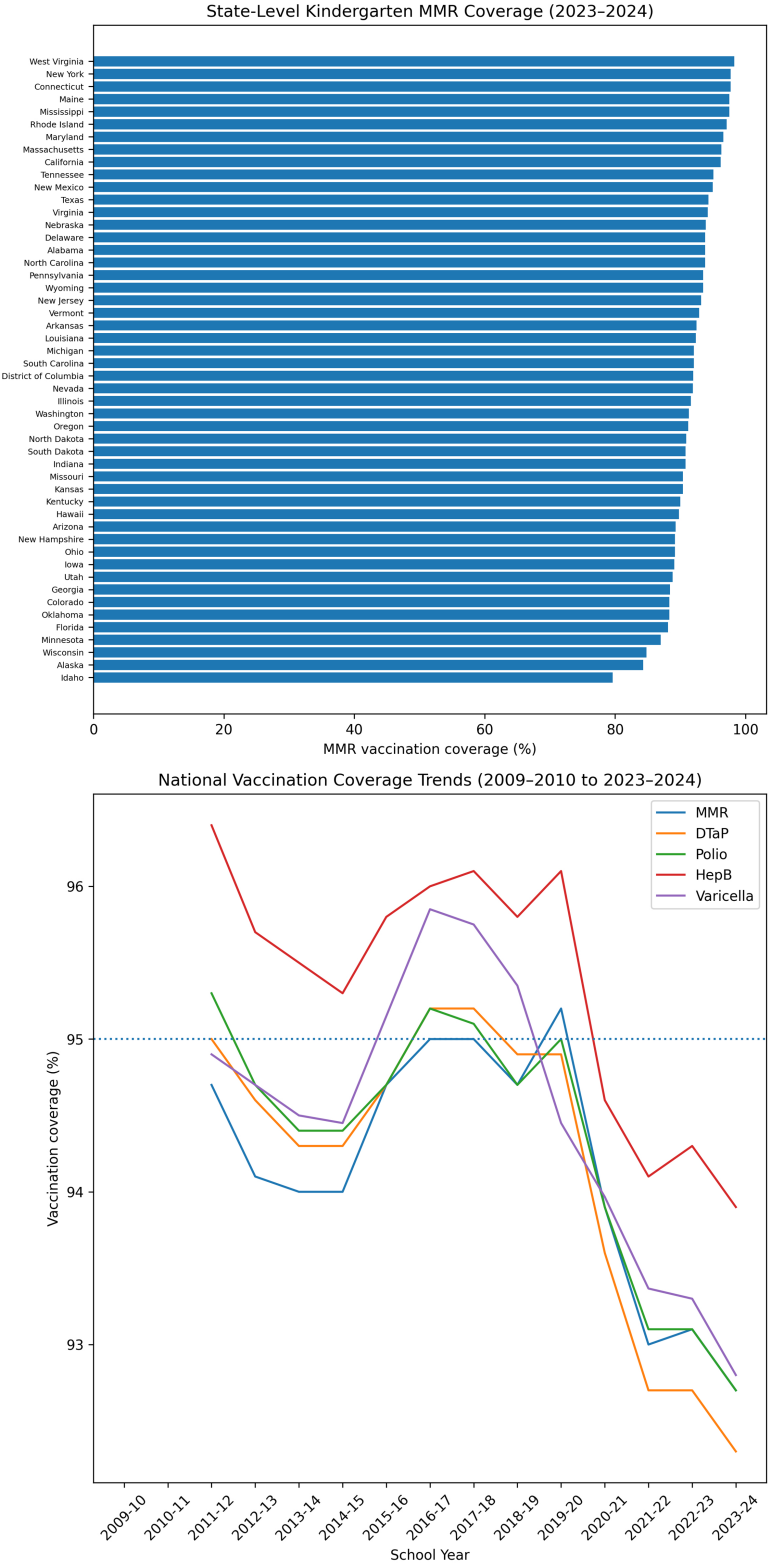
National vaccination trends and state-level MMR coverage, United States. Top panel: Bar chart showing state-level kindergarten measles–mumps–rubella (MMR) vaccination coverage for the 2023–2024 school year, ordered from highest to lowest coverage across U.S. jurisdictions. Bottom panel: Line graph showing national vaccination coverage trends for MMR, diphtheria–tetanus–acellular pertussis (DTaP), polio, hepatitis B, and varicella across school years 2009–2010 through 2023–2024. A dotted horizontal line at 95% indicates the herd immunity threshold for measles. Vaccination coverage declined following the COVID-19 pandemic and has remained below this threshold in recent years.

From the 2016–2017 through 2023–2024 school years, national kindergarten vaccination coverage for all required vaccines remained above 90% but declined gradually over time (7). Measles–mumps–rubella (MMR) coverage fell from approximately 96–97% in the 2018–2019 school year to 92.7% in 2023–2024, marking the first year in more than a decade that coverage for all major childhood vaccines dropped below 93% (Figure 1). Weighted national estimates for the 2024–2025 school year confirmed that coverage stabilized near 92.5%, remaining below the 95% herd immunity threshold (5, 7).

Although coverage remains high by historical standards, this downward shift is epidemiologically meaningful. Modeling studies have shown that measles transmission can resume when coverage falls below approximately 93%–94%, even in previously well-protected populations (8). The current stabilization therefore represents a fragile equilibrium, in which a modest additional decline could permit sustained outbreaks in under-immunized clusters.

### Pandemic disruptions created a lasting plateau rather than a temporary dip

During the 2020–2021 school year, coverage for MMR, diphtheria–tetanus–acellular pertussis, polio, and varicella declined by approximately 1–2 percentage points, coinciding with pandemic-related disruptions to routine care and school-based vaccination enforcement (7, 13). Rather than rebounding after in-person schooling resumed, coverage plateaued at lower levels, suggesting that missed vaccinations during the pandemic period have not been fully recovered (Figure 1).

This persistent shortfall implies that catch-up efforts have not yet closed the vaccination gap that emerged during the COVID-19 pandemic. While routine pediatric visits and school-entry enforcement have largely resumed administratively, the continued lag in vaccination uptake highlights systemic weaknesses in reminder–recall systems and post-pandemic public health outreach (13).

### Exemptions continue to rise, largely reflecting increases in non-medical waivers

**Figure 2:**
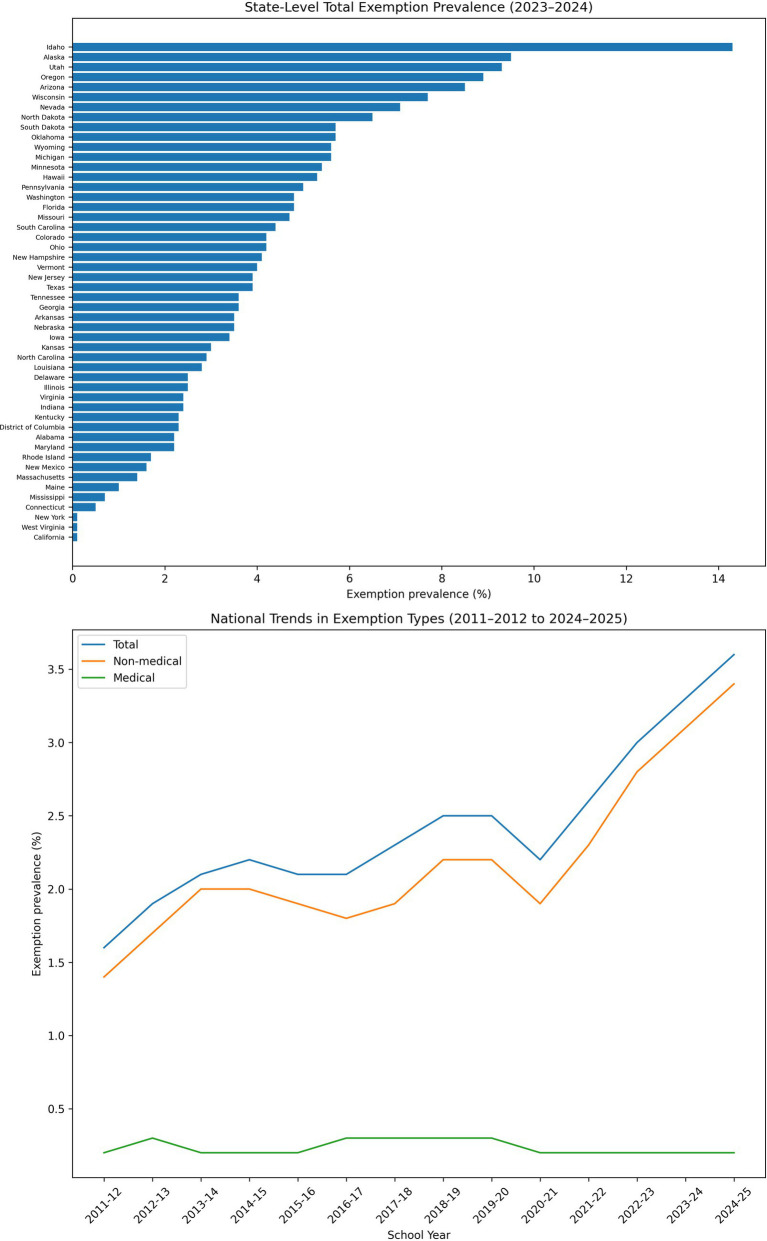
Exemption prevalence and composition among kindergarteners, United States. Top panel: Bar chart showing total exemption prevalence (%) by state for the 2023–2024 school year, ordered from highest to lowest exemption rates across U.S. jurisdictions. Bottom panel: Line graph showing national trends in total, medical, and non-medical exemption prevalence (%) among kindergarteners across school years 2011–2012 through 2024–2025. Non-medical exemptions account for the majority of exemptions and have increased over time, while medical exemptions have remained relatively stable.

The percentage of kindergartners with any documented vaccine exemption increased from 2.5% in the 2018–2019 school year to 3.3% in 2023–2024 and 3.6% in 2024–2025, representing the highest recorded exemption prevalence to date (7). This increase was driven almost entirely by non-medical exemptions, which accounted for more than 90% of all exemptions nationally (Figure 2). In contrast, medical exemptions remained stable at approximately 0.2% nationwide. Fourteen states reported exemption rates exceeding 5%, surpassing the level at which population-level protection against measles cannot be sustained.

The continued growth in non-medical exemptions reflects both legislative trends and administrative practices. Several states have relaxed exemption requirements in recent years, allowing simplified or minimally verified submissions. This policy environment facilitates clustering of undervaccinated children in specific schools or districts, magnifying local outbreak risk despite high statewide averages, consistent with prior findings (14).

### Exemption rates are strongly associated with vaccination coverage

**Figure 3:**
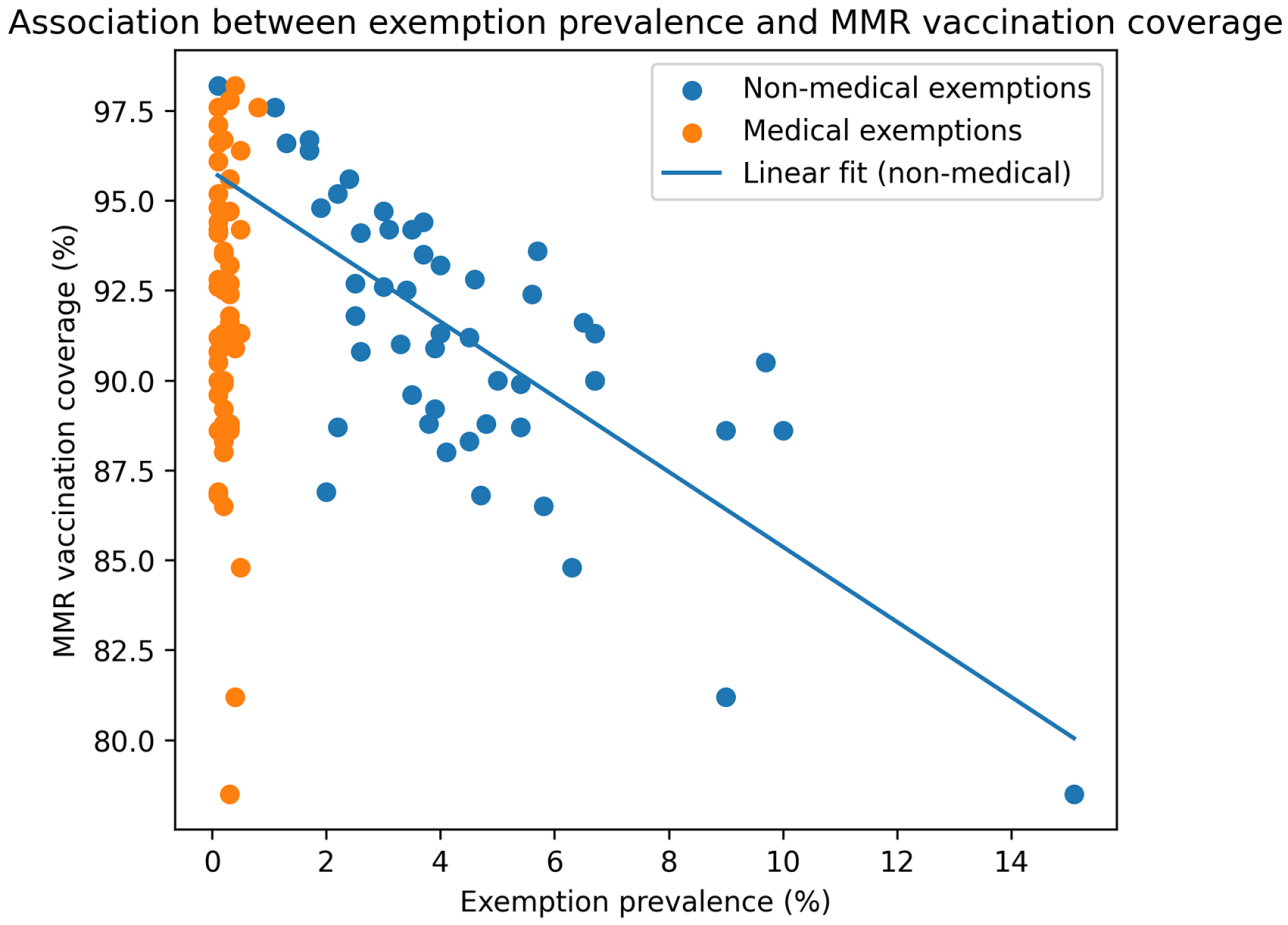
Association between exemption prevalence and MMR vaccination coverage, United States, 2024–2025. Scatterplot illustrating the relationship between kindergarten MMR vaccination coverage (%) and exemption prevalence (%) across U.S. jurisdictions using cross-sectional data from the 2024–2025 school year. Each point represents a jurisdiction-level observation, with non-medical exemptions shown in blue and medical exemptions shown in orange. A fitted linear regression line summarizes the inverse association between exemption prevalence and vaccination coverage. This analysis is observational and does not establish causality.

Higher exemption prevalence was associated with lower MMR vaccination coverage (Figure 3). In a cross-sectional state-level regression analysis using 2024–2025 data (N = 39), each 1% increase in exemption prevalence was associated with an approximately 1.1 percentage-point decrease in MMR coverage (*β* = −1.08, 95% CI: −1.34 to −0.83; SE = 0.13; *p* < 0.001), with exemption prevalence accounting for approximately two-thirds of the observed variation in coverage (R^2^ ≈ 0.66). Full regression outputs are presented in Table 1. Additional computational details underlying the regression model, including derivation of coefficients and model parameters, are provided in Supplementary Table 3.

**Table 1:** Association between exemption prevalence and kindergarten MMR vaccination coverage, United States, 2024–2025.

Variable	Coefficient (*β*)	Standard error	95% Confidence interval	*p*-value
Intercept	96.83	0.70	—	<0.001
Exemption prevalence (%)	−1.08	0.13	−1.34 to −0.83	<0.001

*N* = 39; *R*^2^ = 0.66; *F*-statistic *p* < 0.001. Each 1% increase in exemption prevalence is associated with an approximately 1.08 percentage-point decrease in MMR vaccination coverage.

Descriptive analyses across multiple years demonstrated similar inverse patterns between exemption prevalence and vaccination coverage (Supplementary Figure 1); however, these observations are not derived from the regression model. States with more restrictive exemption policies, such as West Virginia and Mississippi, consistently maintained coverage levels at or above 95%, whereas states permitting broader exemptions, such as Idaho and Alaska, reported substantially lower coverage (7).

While these findings indicate a strong ecological association, they do not establish causality, and the observed relationship may partly reflect unmeasured contextual differences across states, including variation in policy enforcement, local vaccine attitudes, healthcare delivery systems, and school-level administrative practices. Additional correlation analyses and detailed regression outputs are provided in Supplementary Tables 1, 2.

### Coverage disparities between states have widened

**Figure 4:**
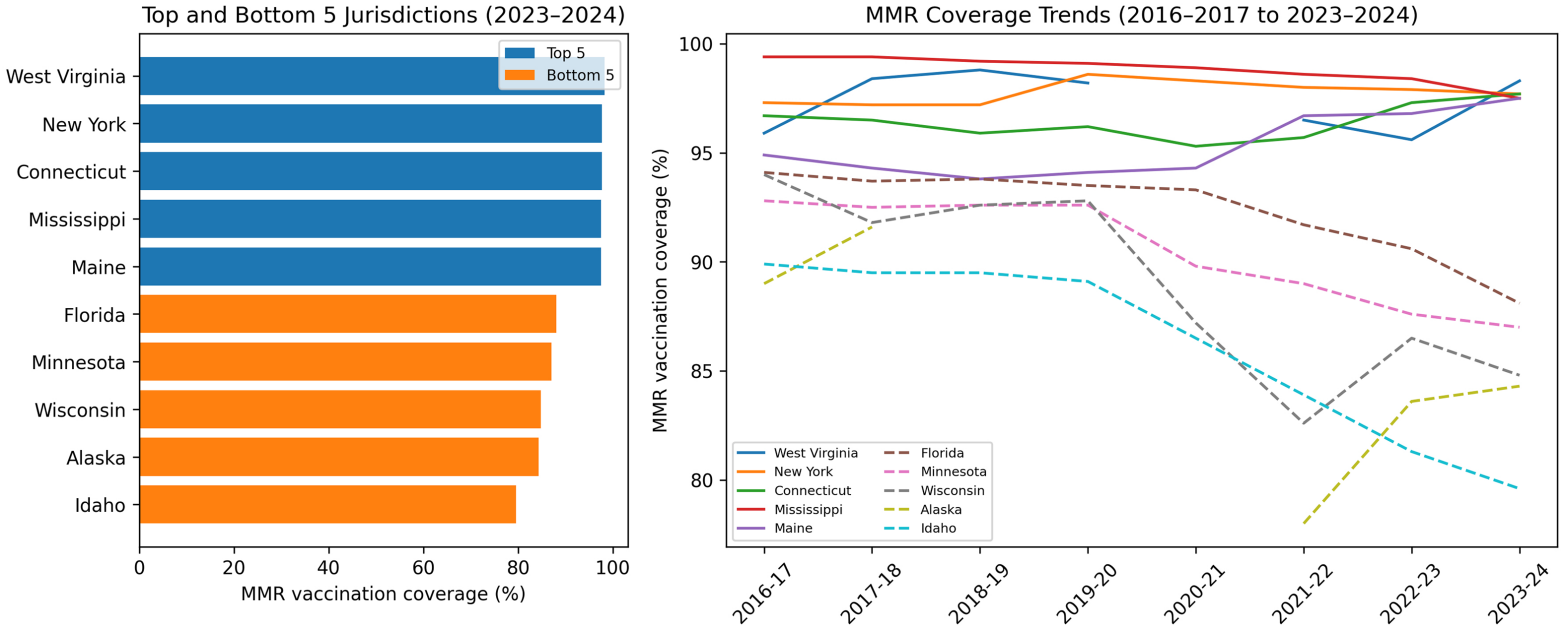
State-level heterogeneity in kindergarten MMR vaccination coverage, United States, 2016–2017 through 2023–2024. Left panel: Horizontal bar chart showing the top five and bottom five U.S. jurisdictions ranked by kindergarten MMR vaccination coverage for the 2023–2024 school year. Top-performing states (West Virginia, New York, Connecticut, Mississippi, and Maine) are displayed at the top, and bottom-performing states (Florida, Minnesota, Wisconsin, Alaska, and Idaho) are displayed below. Right panel: Line graph illustrating trends in MMR vaccination coverage across the same jurisdictions from 2016–2017 through 2023–2024. Top-performing states are shown with solid lines, and bottom-performing states are shown with dashed lines. These panels highlight persistent interstate variation in vaccination coverage over time.

State-level differences in kindergarten vaccination coverage have become more pronounced since 2020 (Figure 4). High-performing states, including West Virginia, New York, Connecticut, Mississippi, and Maine, consistently reported near-universal coverage, whereas Idaho, Alaska, Wisconsin, Minnesota, and Florida reported MMR coverage levels approximately 8–15 percentage points lower (7). These disparities closely mirror variation in exemption policy environments, creating a two-tier system in which state legislation largely determines community protection. A detailed ranking of top- and bottom-performing jurisdictions based on 2024–2025 data is provided in Supplementary Table 4, which reflects the most recent cross-sectional estimates.

This divergence reflects contrasting policy trajectories over the past decade. States that eliminated non-medical exemptions following major outbreaks have maintained resilient vaccination coverage, while others have experienced sustained erosion. In the absence of national standards, herd immunity in the United States remains uneven.

Supplementary Figures 2, 3 provide additional descriptive context regarding state-level variation in vaccination coverage across antigens and comparisons between high- and low-performing states.

## Discussion

Our analysis of national kindergarten vaccination data demonstrates a sustained decline in coverage following the COVID-19 pandemic. Descriptive multi-year data show a persistent downward trend, while cross-sectional analysis of 2024–2025 data demonstrates a strong association between exemption prevalence and vaccination coverage. Despite broad access to vaccines, measles–mumps–rubella (MMR) vaccination coverage has plateaued near 92%–93%, remaining below the 95% threshold required to prevent measles transmission (7). This plateau indicates that post-pandemic recovery has been incomplete and suggests that education and outreach alone may not be sufficient to restore population-level protection.

### Exemptions as a policy lever

Non-medical exemptions are strongly associated with variation in state-level vaccination coverage and represent a measurable policy-relevant factor. Higher exemption prevalence was associated with lower MMR vaccination coverage, accounting for a substantial proportion of interstate variation. These findings suggest that state exemption policies are an important structural factor shaping differences in herd immunity across states. States that tightened exemption policies, including California and New York, experienced measurable increases in vaccination coverage following legislative reform (9, 10), whereas states that maintained permissive exemption policies have continued to experience erosion in coverage.

Efforts to modify or eliminate non-medical vaccine exemptions often face substantial legal, political, and institutional barriers. Vaccination requirements for school entry are established through state legislation, and changes to exemption policies therefore require legislative action that can be influenced by political polarization and organized advocacy groups. Opposition to tightening exemption laws frequently invokes arguments related to individual autonomy, parental rights, and religious freedom, and legislative proposals may encounter constitutional scrutiny depending on how exemptions are structured under state law. In addition, organized campaigns opposing vaccination mandates and the spread of vaccine-related misinformation through social media and advocacy networks can shape public opinion and influence legislative debate. Even when policy changes are enacted, implementation may be uneven, as enforcement of school-entry vaccination requirements depends on administrative capacity within state health departments, local public health agencies, and school systems. These legal, political, and administrative dynamics help explain why exemption policies vary widely across states and why efforts to strengthen vaccination requirements can be difficult to enact and sustain over time.

The measles resurgence observed in 2025, which resulted in more than 2,200 confirmed cases across multiple U.S. jurisdictions, highlights how erosion of vaccination coverage can rapidly translate into widespread transmission (11). Outbreaks concentrated in states with rising exemption rates illustrate how relatively small declines in coverage can facilitate community spread.

### State heterogeneity and targeted interventions

National averages obscure widening disparities between states. Some jurisdictions, including West Virginia and Mississippi, consistently sustain coverage above 95%, whereas others—notably Idaho and Alaska—remain 10–15 percentage points below that threshold (7). These differences closely align with variation in exemption policies, suggesting that national herd immunity depends on localized enforcement and implementation.

Targeted interventions at the state and local level may be an important component of efforts to improve vaccination coverage. Strengthening school-entry vaccination verification, ensuring consistent standards across public and private schools, and supporting catch-up vaccination programs in low-coverage areas represent practical and actionable strategies for health departments.

### Public health implications

Because exemption laws are modifiable at the state level, legislative reform represents a potentially important intervention to improve population-level immunity. Restricting non-medical exemptions, standardizing documentation requirements, and maintaining transparent exemption reporting are policy approaches that may be associated with higher vaccination coverage. Continued surveillance should monitor exemption trends, evaluate the impact of policy changes, and integrate coverage data with outbreak monitoring systems. Sustained coordination between federal and state agencies will be essential to maintain accountability and ensure that exemption policies support, rather than undermine, public health goals.

### Limitations

This study has several limitations. First, it relies on aggregate, state-reported surveillance data that vary in completeness and methodology; some states report census-based data, whereas others rely on sampling, which may introduce measurement bias. Second, the ecological design precludes causal inference; although exemption prevalence and vaccination coverage are strongly associated, other contextual factors—such as enforcement practices or community-level attitudes—may also contribute.

Third, the analysis employs a bivariate regression model to estimate the association between exemption prevalence and MMR vaccination coverage, rather than to construct a comprehensive causal model of vaccination uptake. These variables are closely linked within school-entry immunization reporting systems, as exemptions represent the primary mechanism through which children remain unvaccinated despite vaccination requirements. Consequently, exemption prevalence serves as a direct indicator of behavioral and policy dynamics affecting coverage. The analysis uses aggregated jurisdiction-level data from the CDC SchoolVaxView system, where vaccination coverage and exemption prevalence are reported consistently across states. Comparable measures for socioeconomic conditions, demographics, healthcare infrastructure, or school characteristics are not uniformly available within the same dataset and would require substantial data harmonization, potentially introducing measurement inconsistencies. Given these constraints and the relatively small analytic sample, a parsimonious specification provides a clear and interpretable estimate of the observed association. Alternative modeling approaches for proportion outcomes, such as quasi-binomial or beta regression models, could also be considered; however, linear regression was selected for its interpretability and its direct expression of percentage-point changes relative to herd immunity thresholds.

Fourth, the analysis focuses on vaccination status at kindergarten entry and does not capture delays that may later be corrected. Finally, the regression models and visualizations describe associations rather than underlying mechanisms. Nevertheless, the consistency of these findings across multiple years and jurisdictions strengthens confidence in the observed relationships. Future studies incorporating larger datasets, multivariable approaches, or multi-level designs could further examine the influence of broader contextual factors—including socioeconomic conditions, demographic composition, healthcare access, and school-level policies—on vaccination coverage.

## Conclusion

Eight years of CDC kindergarten surveillance data indicate that U.S. childhood vaccination coverage, while still high, has become increasingly fragile. Following pandemic-related declines, national coverage has not fully rebounded, and rising non-medical exemptions continue to erode herd immunity. States that limit or eliminate non-medical exemptions sustain higher coverage, whereas those allowing broad exemptions remain persistently below the 95% target. These findings suggest that policy environments may play an important role in shaping community protection against vaccine-preventable diseases, alongside other factors such as access, healthcare delivery, and public attitudes toward vaccination.

Sustained progress will likely require more than communication and education alone and may also depend on aligning state policies with public health objectives (15). Strengthening enforcement of school-entry vaccination requirements, maintaining transparent exemption reporting, and expanding community-based catch-up programs represent actionable strategies to restore population-level protection. Continued surveillance and accountability will be essential to ensure that herd immunity—one of public health’s most reliable safeguards—does not continue to erode.

## Data Availability

The data used in this study are publicly available from the Centers for Disease Control and Prevention (CDC) Open Data portal. Specifically, we used the “Vaccination Coverage and Exemptions among Kindergartners” dataset, maintained by the CDC National Center for Immunization and Respiratory Diseases (NCIRD) as part of the School Vaccination Assessment Program (SchoolVaxView). Repository: CDC Open Data Portal Direct link: https://data.cdc.gov/. Dataset identifier (accession number): ijqb-a7ye. All data are in the public domain and were accessed without restriction.
