# Supplementary material for: Non-medical exemptions and state-level variation in kindergarten MMR vaccination coverage, United States, 2016–2024
Source: Front Public Health. 2026 Apr 30;14:1795927. doi: 10.3389/fpubh.2026.1795927 (PMC13171547; doi:10.3389/fpubh.2026.1795927)
Supplement: Supplementary file 1 [file Data_Sheet_1.docx]

**Supplementary Methods**

Regression diagnostics were conducted for the 2024–2025 cross-sectional model using the 39-jurisdiction analytic sample after excluding provisional values and jurisdictions with NR. A Ramsey RESET test was used to evaluate functional form misspecification; the result was not statistically significant (F = 0.015, p = 0.903), exceeding the conventional significance threshold of p > 0.05, thereby supporting the linear specification. Residual normality was assessed using the Shapiro–Wilk test, where values of p ≤ 0.05 indicate deviation from normality.

The test suggested some departure from normality (W = 0.922, p = 0.010). However, the primary objective of the analysis is to estimate the association between exemption prevalence and vaccination coverage, for which ordinary least squares estimates remain unbiased and consistent even when residuals deviate from normality. Homoscedasticity was evaluated using the Breusch–Pagan test, where p > 0.05 indicates no evidence of heteroskedasticity; the test was not significant (χ² = 0.687, p = 0.407).

Independence of residuals was assessed using the Durbin–Watson statistic, where values near 2 indicate no autocorrelation and values between approximately 1.5 and 2.5 are generally considered acceptable. The observed statistic (DW = 1.603) falls within this range, suggesting no severe autocorrelation in this cross-sectional sample. Although the residual normality test indicates some deviation, the absence of heteroskedasticity or strong autocorrelation suggests that the regression results remain informative for examining the observed relationship. Nonetheless, this deviation from normality is acknowledged as a limitation, and future analyses using larger datasets could employ robust regression or nonparametric approaches to further assess the stability of the findings.

**Supplementary Results**

**Regression Results and Sample Construction**

A simple ordinary least squares (OLS) regression was estimated to examine the relationship between MMR vaccination coverage (%) and exemption prevalence (%) across U.S. jurisdictions in the 2024–2025 school year. Jurisdictions with missing values (“NR”) and jurisdictions with provisional estimates marked with “?” in the CDC SchoolVaxView dataset were excluded from the analysis to ensure that the regression relied only on finalized and fully reported observations. Specifically, the excluded jurisdictions included Colorado, Florida, Georgia, Illinois, Iowa, Minnesota, Mississippi, Missouri, New Hampshire, and New Jersey (all marked with provisional values), as well as Montana and West Virginia, which were reported as NR (not reported). After applying these exclusions, the final analytic sample consisted of 39 jurisdictions.

Using this filtered dataset, the estimated regression equation was:

$$\hat{Y}=96.86-1.08X$$

where $Y$ represents MMR vaccination coverage (%) and $X$represents exemption prevalence (%). The coefficient for exemption prevalence indicates a negative and statistically significant relationship between exemptions and MMR vaccination coverage. Substantively, each one-percentage-point increase in exemption prevalence is associated with an approximately 1.08 percentage-point decrease in MMR vaccination coverage.

The model demonstrates a strong explanatory relationship between exemption prevalence and vaccination coverage. The regression yields an $R^{2}$of 0.6638, meaning that approximately 66.4% of the variation in MMR vaccination coverage across jurisdictions is explained by exemption prevalence alone. This value closely matches the reported $R^{2}\approx0.662$, with the small difference attributable to rounding.

The $R^{2}$value was verified through the standard decomposition of variance:

$$R^{2}=\frac{SSR}{SST}=1-\frac{SSE}{SST}$$

Using the filtered sample of 39 jurisdictions, the regression produced a total sum of squares (SST) of 689.98, a regression sum of squares (SSR) of 457.98, and a residual sum of squares (SSE) of 231.99, yielding:

$$R^{2}=\frac{457.98}{689.98}=0.6638$$

Because this is a simple linear regression with a single predictor, $R^{2}$ is equivalent to the square of the Pearson correlation between exemption prevalence and vaccination coverage, which likewise equals 0.6638 in the analytic sample. Additionally, the value is internally consistent with the regression test statistics: with $N=39$observations and one predictor, the model produces F(1,37) ≈ 73.04, and since $F=t^{2}$in simple regression, the squared t-statistic for the slope coefficient yields the same value, confirming the accuracy of the reported $R^{2}$.

Overall, the results demonstrate a strong inverse relationship between exemption prevalence and MMR vaccination coverage, indicating that jurisdictions with higher exemption rates tend to have substantially lower vaccination coverage.

**Cross-Correlation and OLS Regression Analysis (2024–25)**

**Interpretation of Regression Analysis**

The correlation matrix reveals a strong negative relationship (r = –0.814) between MMR coverage and exemption rates across U.S. states, indicating that higher exemption prevalence is associated with lower vaccination coverage.

With an implied R² ≈ 0.66, the model explains roughly two-thirds of the variation in MMR coverage across states.

Overall, these findings indicate a strong inverse association between exemption prevalence and vaccination coverage across jurisdictions. Jurisdictions with higher exemption rates—such as Idaho and Alaska—tend to have substantially lower vaccination coverage, whereas those with lower exemption rates maintain higher coverage levels. These patterns are consistent with exemption prevalence being an important factor associated with variation in vaccination coverage, although causal relationships cannot be established from this analysis.

**Supplementary Table 1. Correlation Matrix for MMR Vaccination Coverage and Exemption Prevalence Across U.S. Jurisdictions, 2024–2025**

| **Variable** | **MMR Coverage (%)** | **Exemption Rate (%)** |
| --- | --- | --- |
| MMR Coverage (%) | 1 | –0.814 |
| Exemption Rate (%) | –0.814 | 1 |

**Supplementary Table 2. Ordinary Least Squares Regression Results for the Association Between Exemption Prevalence and Kindergarten MMR Vaccination Coverage, 2024–2025**

| **Variable** | **Coefficient (β)** | **Std. Error** | **t-Statistic** | **P-Value** |
| --- | --- | --- | --- | --- |
| Constant (α) | 96.83 | 0.7 | 138.68 | < 0.001 |
| Exemption Rate (%) | –1.08 | 0.13 | –8.63 | < 0.001 |

- R² = 0.66, meaning 66% of the variance in MMR coverage across states is explained by exemption rates.
- F-statistic: highly significant (*p* < 0.001).
- Each 1% increase in exemptions corresponds to an estimated 1.08 percentage-point decrease in MMR coverage.

**Regression Computation and Model Summary (2024–25)**

This section outlines the computation steps used to derive the regression coefficients and model parameters.

Model: Yᵢ = α + βXᵢ + εᵢ, where Yᵢ = MMR coverage (%) and Xᵢ = exemption rate (%).

**Supplementary Table 3. Step-by-Step Computation of Regression Parameters for the Association Between Exemption Prevalence and MMR Coverage**

| **Computation Step** | **Value / Formula** |
| --- | --- |
| Sample means | Ȳ = 92.7, X̄ = 3.7 |
| Sample standard deviations | s_Y = 5.2, s_X = 3.2 |
| Correlation coefficient | r = –0.8137 |
| Slope (β) | β = r × (s_Y / s_X) = –1.08 |
| Intercept (α) | α = Ȳ – βX̄ = 96.7 |
| Regression equation | Ŷ = 96.83 – 1.08X |
| Coefficient of determination | R² = r² = 0.66 |

**Updated 2024–25 Weighted Results**

Using the CDC SchoolVaxView weighted state-level counts (2024–25), the national MMR coverage among U.S. kindergarteners reached 92.5%, while 3.6% of children were exempt from one or more required vaccines. This marks a 0.3-percentage-point increase in exemptions compared with the previous year, reaffirming a persistent shortfall in achieving the 95% herd immunity benchmark.

Weighted analyses reveal pronounced interstate variation. Coverage exceeded 96% in several Northeastern states—Connecticut (98.2%), New York (97.8%), and Maine (97.6%)—indicating strong compliance and effective enforcement of immunization mandates. In contrast, multiple western and midwestern states reported substantially lower coverage, led by Idaho (78.5%), Alaska (81.2%), and Wisconsin (84.8%), each with comparatively higher exemption rates.

These disparities highlight substantial variation in vaccination coverage across jurisdictions and provide additional context for understanding differences in population-level protection.

**Supplementary Table 4. Top and Bottom U.S. Jurisdictions by Kindergarten MMR Vaccination Coverage and Exemption Prevalence, 2024–2025**

| **Rank** | **Category** | **State** | **MMR Coverage (%)** | **Exemptions (%)** |
| --- | --- | --- | --- | --- |
| 1 | Top 5 | Connecticut | 98.2 | 0.4 |
| 2 |  | New York (including NYC) | 97.8 | 0.3 |
| 3 |  | Maine | 97.6 | 0.8 |
| 4 |  | Rhode Island | 96.7 | 1.9 |
| 5 |  | Massachusetts | 96.6 | 1.5 |
| 1 | Bottom 5 | Idaho | 78.5 | 15.4 |
| 2 |  | Alaska | 81.2 | 9.4 |
| 3 |  | Wisconsin | 84.8 | 7.6 |
| 4 |  | Minnesota | 86.5 | NR* |
| 5 |  | Kentucky | 86.9 | 2.2 |

*NR: Not reported

**Supplementary Figure 1. Association between exemption prevalence and MMR vaccination coverage across U.S. jurisdictions, 2016–2024**

Scatterplot illustrating the relationship between kindergarten MMR vaccination coverage (%) and total exemption prevalence (%) across U.S. jurisdictions using pooled state-year observations from the 2016–2017 through 2023–2024 school years. Each point represents a jurisdiction-year observation. A fitted linear trend line summarizes the overall inverse relationship between exemption prevalence and vaccination coverage. This figure is descriptive and includes repeated observations from the same jurisdictions across multiple years; it was not used for the primary regression analysis.

**Supplementary Figure 2. Distribution of state-level kindergarten vaccination coverage by antigen, United States, 2023–2024**

Boxplots display the distribution of vaccination coverage (%) across U.S. jurisdictions for measles–mumps–rubella (MMR), diphtheria–tetanus–acellular pertussis (DTaP), polio, hepatitis B, and varicella vaccines during the 2023–2024 school year. Each box represents the interquartile range with the median indicated, and whiskers reflect the range of observed values. Variation across states highlights differences in population-level vaccination coverage.

**Supplementary Figure 3. Comparison of vaccination coverage across selected high- and low-performing states, United States, 2023–2024**

Bar charts display vaccination coverage (%) for measles–mumps–rubella (MMR), diphtheria–tetanus–acellular pertussis (DTaP), polio, hepatitis B, and varicella vaccines across the five states with the highest and lowest MMR vaccination coverage in the 2023–2024 school year (states ranked by MMR coverage). Differences across states are consistent across vaccines, illustrating broad variation in vaccination uptake across jurisdictions.
