# Supplementary figures and images for: Non-medical exemptions and state-level variation in kindergarten MMR vaccination coverage, United States, 2016–2024
Source: Front Public Health. 2026 Apr 30;14:1795927. doi: 10.3389/fpubh.2026.1795927 (PMC13171547; doi:10.3389/fpubh.2026.1795927)

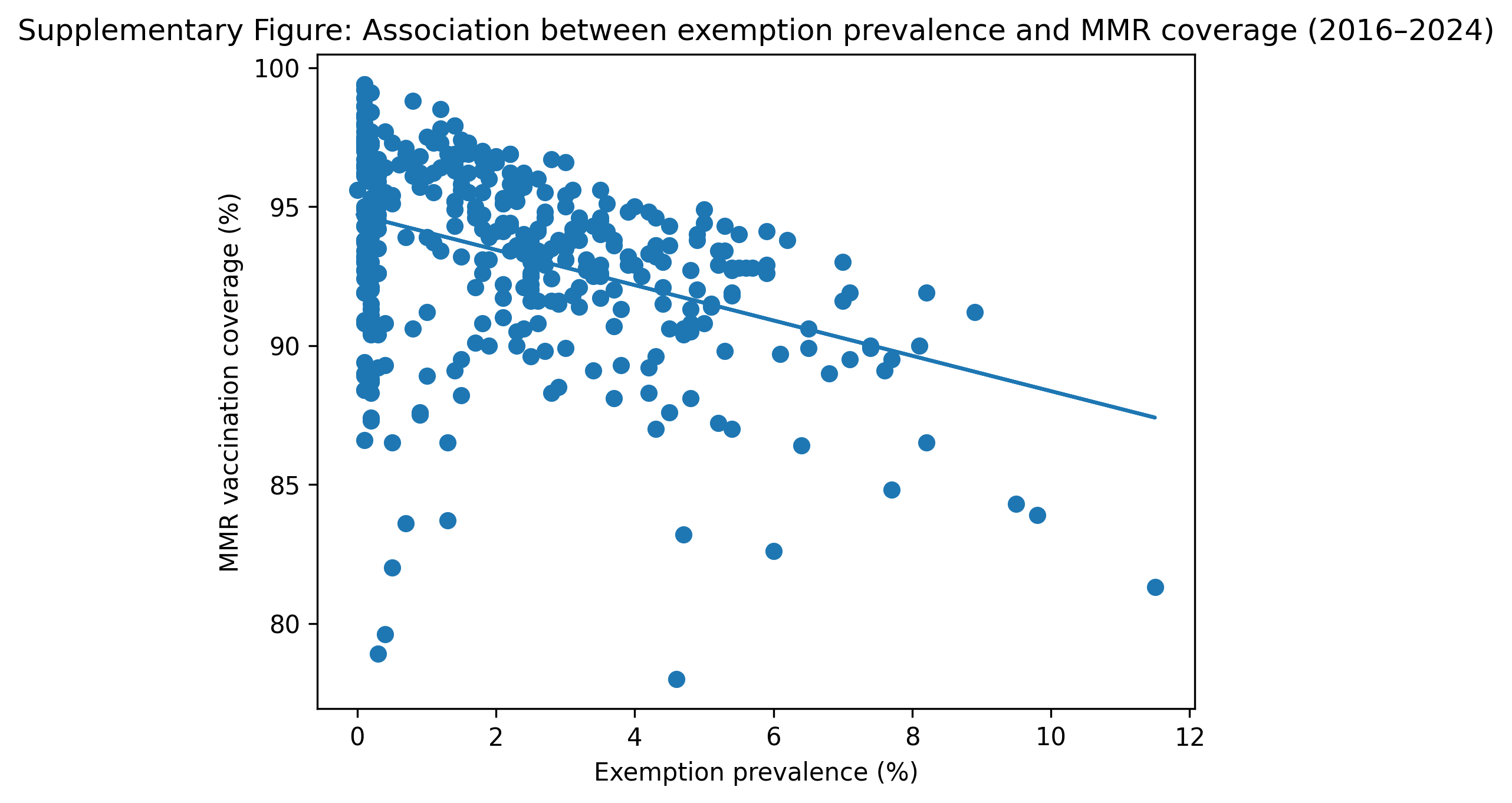

Supplement: Supplementary file 2 [file Image_1.jpeg]

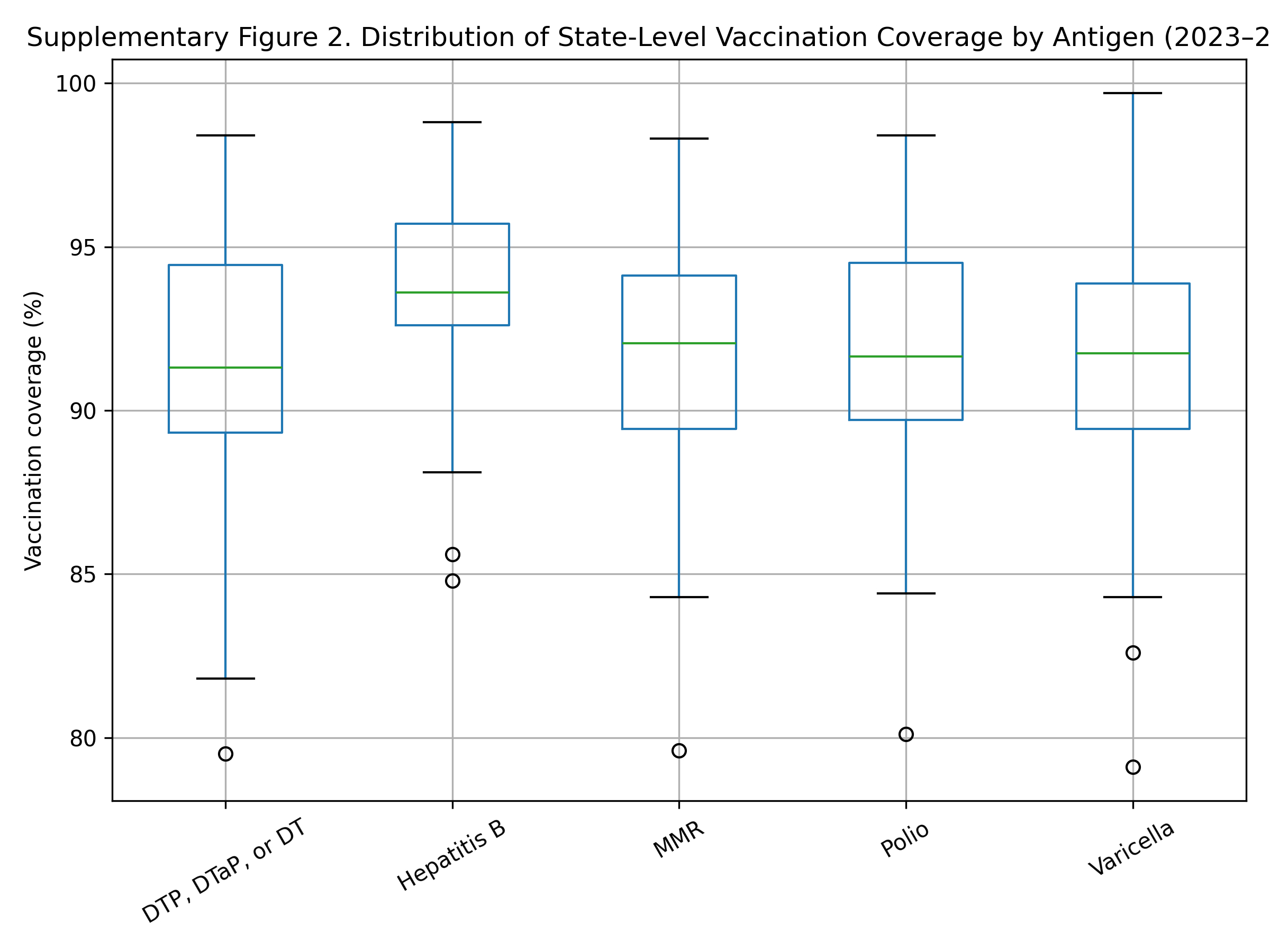

Supplement: Supplementary file 3 [file Image_2.jpeg]

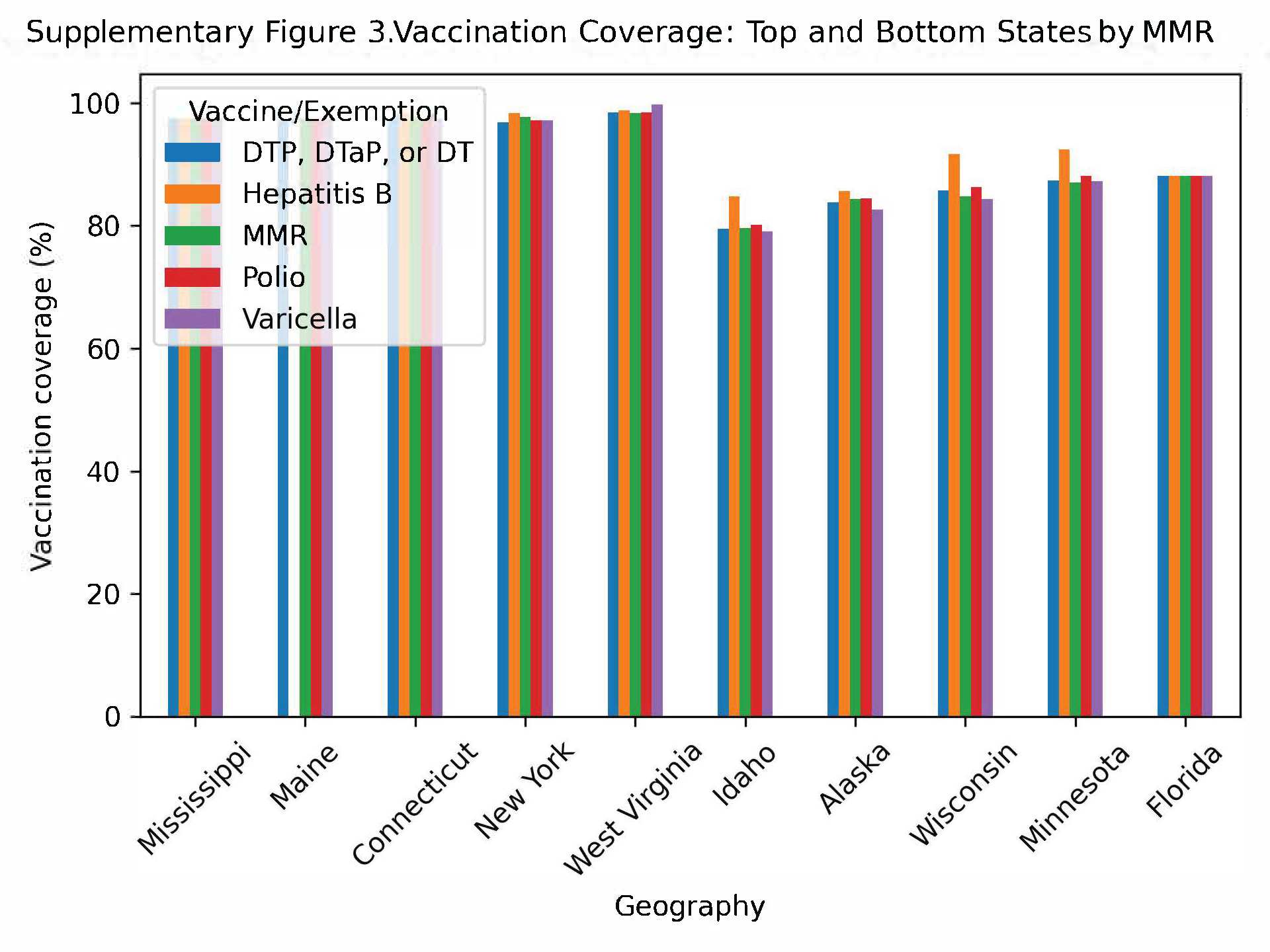

Supplement: Supplementary file 4 [file Image_3.png]
